# Supplementary material for: An epidemiological surveillance of hand foot and mouth disease in paediatric patients and in community: A Singapore retrospective cohort study, 2013–2018
Source: PLoS Negl Trop Dis. 2021 Feb 10;15(2):e0008885. doi: 10.1371/journal.pntd.0008885 (PMC7901731; doi:10.1371/journal.pntd.0008885)
Supplement: S1 Text — (DOCX) [file pntd.0008885.s001.docx]

After including the Singaporean sequences with the reference alignment, the temporal structure of the sequences was assessed using TreeTime (least-squares method) [32]. There is a positive but diffuse correlation between genetic divergence and sampling dates ($R^{2}=0.28$).
