# Supplementary figures and images for: An epidemiological surveillance of hand foot and mouth disease in paediatric patients and in community: A Singapore retrospective cohort study, 2013–2018
Source: PLoS Negl Trop Dis. 2021 Feb 10;15(2):e0008885. doi: 10.1371/journal.pntd.0008885 (PMC7901731; doi:10.1371/journal.pntd.0008885)

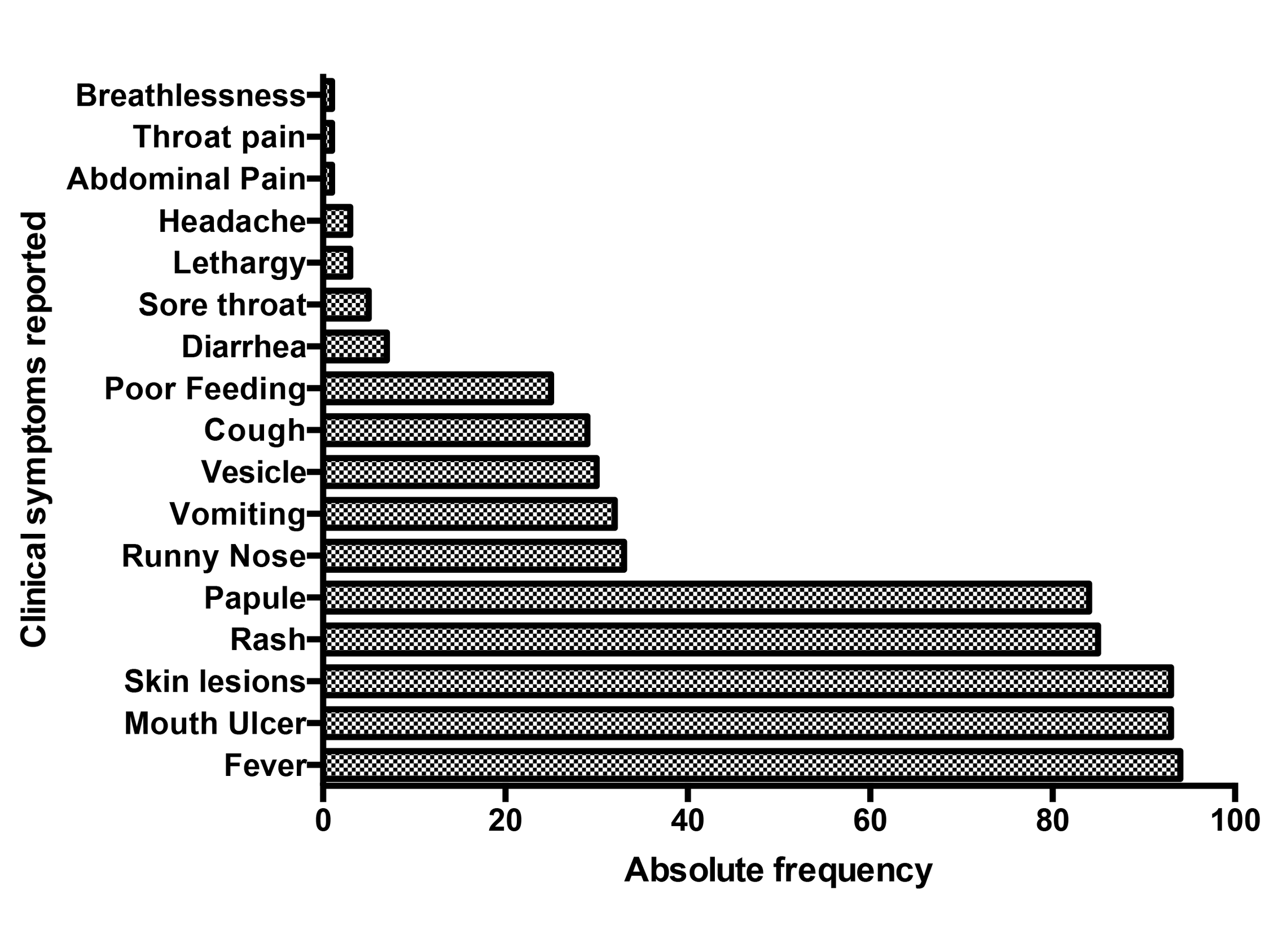

Supplement: S1 Fig — These clinical symptoms were recorded from the pediatric patients during admission and several symptoms were presented by the majority of the patients, including fever, mouth ulcer, skin lesion, rashes and papules. (TIF) [file pntd.0008885.s003.tif]

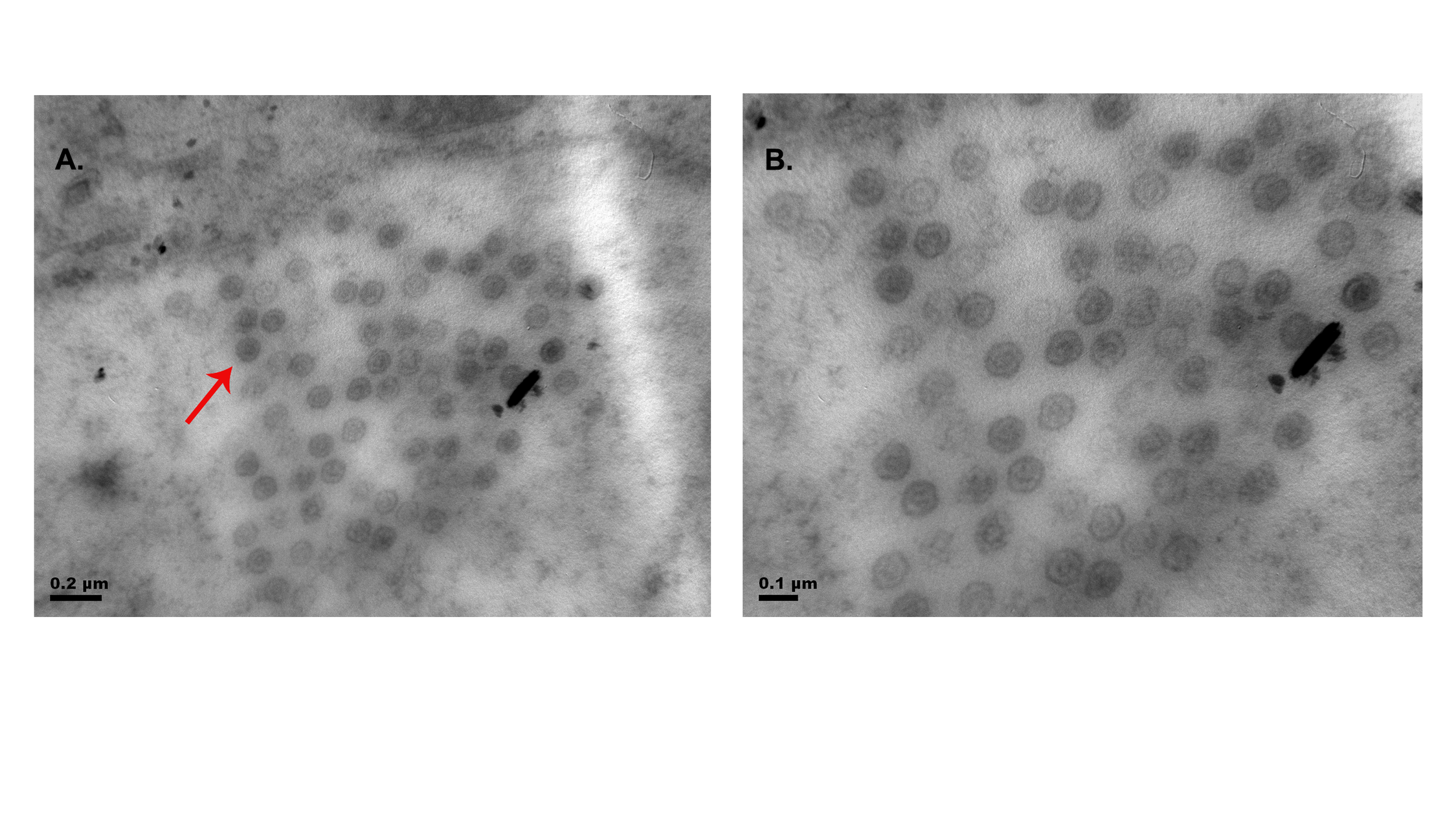

Supplement: S2 Fig — Isolated HSV1 viruses were used to infect RD cell at MOI of 1. Infected cells were fixed at 16 hours post infection and processed for TEM analysis. (TIF) [file pntd.0008885.s004.tif]

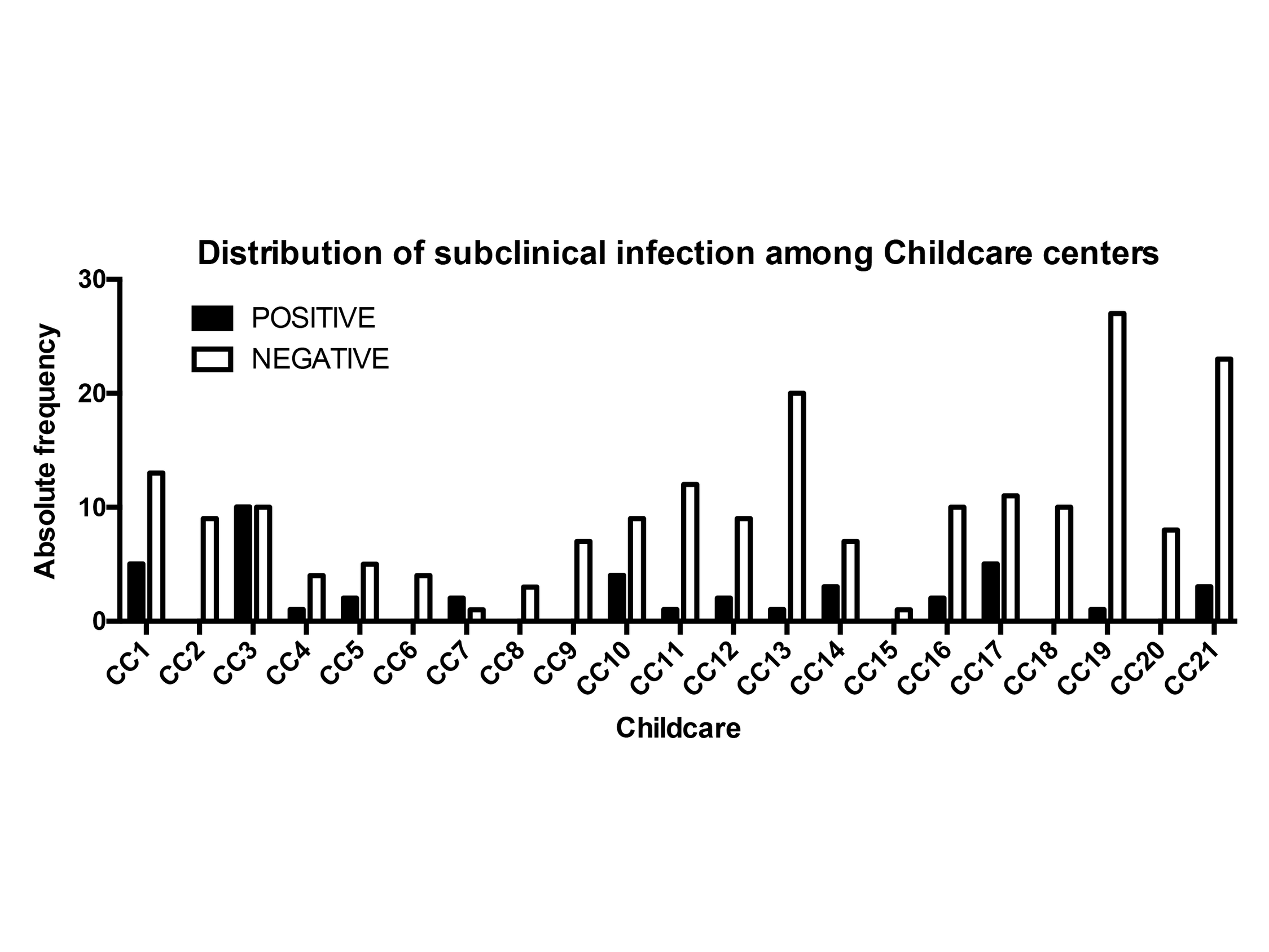

Supplement: S3 Fig — Based on the Pan-Entero RT-PCR assay, the subclinical infections were dispersed across all the childcare center sampled, revealing a basal level of subclinical infection across most childcare centers. (TIF) [file pntd.0008885.s005.tif]

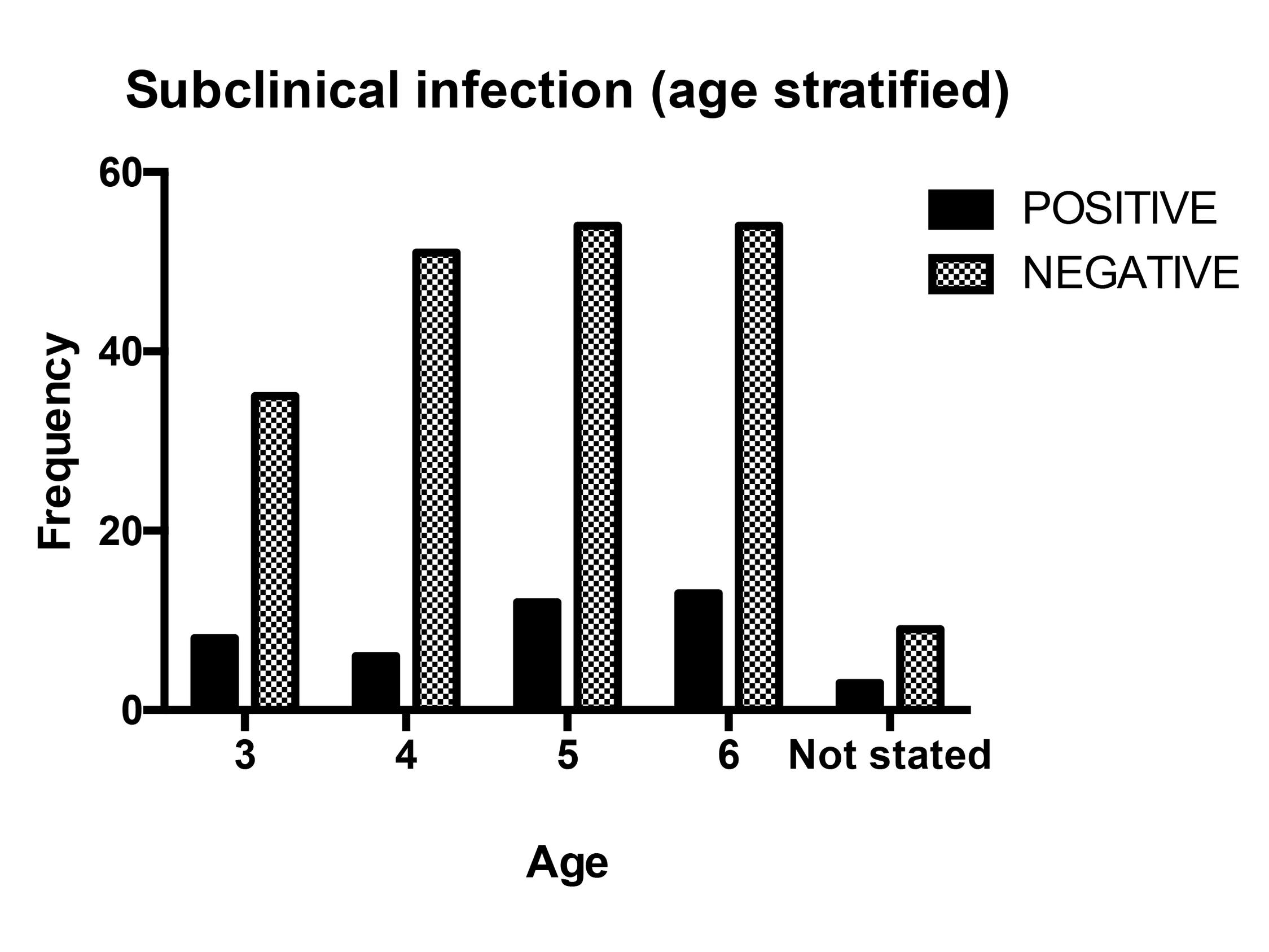

Supplement: S4 Fig — Subclinical infections were stratified according to the different ages of the children. (TIF) [file pntd.0008885.s006.tif]
