# Supplementary material for: An epidemiological surveillance of hand foot and mouth disease in paediatric patients and in community: A Singapore retrospective cohort study, 2013–2018
Source: PLoS Negl Trop Dis. 2021 Feb 10;15(2):e0008885. doi: 10.1371/journal.pntd.0008885 (PMC7901731; doi:10.1371/journal.pntd.0008885)
Supplement: Attachment — Submitted filename: PNTD-D-20-01211.docx [file pntd.0008885.s007.docx]

**PNTD-D-20-01211: Epidemiology of Hand Foot and Mouth Disease in Paediatric Patients and in Community: A Retrospective Cohort Study, 2013-2018**

The authors of this manuscript studied HFMD and subclinical cases from the years 2013 to 2018 in Singapore. Their findings showed that majority of the samples collected were cased by coxsackievirus A6, while a smaller percentage being caused by EV-A71, CV-A16, CV-A2, and CV-A10. They also compared the positive testing rate between samples collected via throat swab and from saliva. The findings indicated that samples from throat swabs had a higher positive rate in virus detection compared to saliva samples. The authors also reported that 17.1% tested positive for enterovirus in saliva among healthy children in daycare centers. A few points are mentioned below that are needed for clarification:

1. In page 8 line 155, understandably a full recovery means no symptoms and fever present. However, does the partial recovery status mean that some of the symptoms such as mouth ulcers or rashes are still present or just a slight fever?
2. Page 9 line 188. A typo was written as ‘throat-swan’.
3. Page 10 line 194. The abbreviation for CV-A16 was used inconsistently and was written as CA16.
4. Page 11 Line 225. Reference style inconsistency.
5. Page 22. Please clarify the grading for characteristics in Table 2.
6. Page 27 Figure 3. The text in this figure is difficult to distinguish, even in the PDF file. Is there a clearer figure to replace this one?
