# Supplementary material for: An epidemiological surveillance of hand foot and mouth disease in paediatric patients and in community: A Singapore retrospective cohort study, 2013–2018
Source: PLoS Negl Trop Dis. 2021 Feb 10;15(2):e0008885. doi: 10.1371/journal.pntd.0008885 (PMC7901731; doi:10.1371/journal.pntd.0008885)
Supplement: Attachment — Submitted filename: PNTD-D-20-01211.docx [file pntd.0008885.s008.docx]

**PNTD-D-20-01211: Epidemiology of Hand Foot and Mouth Disease in Paediatric Patients and in Community: A Retrospective Cohort Study, 2013-2018**

The authors of this manuscript studied HFMD and subclinical cases from the years 2013 to 2018 in Singapore. Their findings showed that majority of the samples collected were cased by coxsackievirus A6, while a smaller percentage being caused by EV-A71, CV-A16, CV-A2, and CV-A10. They also compared the positive testing rate between samples collected via throat swab and from saliva. The findings indicated that samples from throat swabs had a higher positive rate in virus detection compared to saliva samples. The authors also reported that 17.1% tested positive for enterovirus in saliva among healthy children in daycare centers. A few points are mentioned below that are needed for clarification:

1. In page 8 line 155, understandably a full recovery means no symptoms and fever present. However, does the partial recovery status mean that some of the symptoms such as mouth ulcers or rashes are still present or just a slight fever?

**Thank you for your clarification. Partial recovery status in this context meant discharge from hospital with the presence of either one or more WHO defined HFMD symptom including: fever; sore throat; blister-like lesions on the tongue, gums and inside of the cheeks; red rash without itching but sometimes with blistering, on the palms, soles and sometimes the buttocks. The discharge of patient could have happened by an attending pediatrician who deemed patient condition to be safe to rest at home or by a guardian. We have added the explanation on line 172-173, “There were no fatal or severe cases of HFMD reported with 92.3% (n = 96) having a full recovery and 7.7% (n = 8) were discharged with partial recovery status, in which the patient is discharged with either one or more WHO defined HFMD symptom.”**

2. Page 9 line 188. A typo was written as ‘throat-swan’.

**Thank you for catching the typo; we have corrected the spelling.**

3. Page 10 line 194. The abbreviation for CV-A16 was used inconsistently and was written as CA16.

**Thank you again for catching the typo; we have corrected the spelling.**

4. Page 11 Line 225. Reference style inconsistency.

**We have corrected the style.**

5. Page 22. Please clarify the grading for characteristics in Table 2.

**In Table 2, factors such as “Age”, “Race”, “Gender” and “Previous HFMD History” were stratified between asymptomatic (subclinical) and healthy cohort.**

6. Page 27 Figure 3. The text in this figure is difficult to distinguish, even in the PDF file. Is there a clearer figure to replace this one?

**We have made changes to the figure to make it clearer and submitted the high-resolution TIFF files.**

Please address all reviewers’ and the following comments:

1. Please improve the clarity of the figures and increase the font size of the labels/text.

**We have amended the figures to be clearer**.

2. Methods, please clarify how the patients were recruited. Were there only 104 HFMD patients over 5 years in the KK Women’s and Children’s Hospital?

**There were several hundreds of HFMD hospitalized cases in Singapore typically in a given year. However, recruitment of patient for consent so samples could be used in medical research was proved to be extremely challenging throughout the study. We postulate that it could be due to several reasons including the age of patient, guardians not willing to disclose personal data outside of the hospital and guardian(s) refusing to participate due to the lethargic nature of the disease inflicted on patient.**

3. Tables 1 & 2 and Figure 5, please change “TRUE”/”FALSE” to “Yes”, “No”

**We have made changes accordingly.**

4. Discuss the use of VP1 sequence on the interpretation of results

**We have added the following paragraph into discussion (line 318-322).**

**“VP1 protein of enteroviruses contains a number of neutralization domains which correspond with enterovirus serotypes and consequently the phylogenetic lineage. VP1 sequences are therefore widely used as molecular determinant of serotypes and genotypes in the field. It has been well established that VP1 and VP4 sequences of enteroviruses are well suited for serotyping and genotyping of enteroviruses [1].”**

**In this study we used partial VP1 sequence of enteroviruses to identify the serotype and genotype of enteroviruses detected. However, going forward, the limitation of the use of VP1 sequence is discussed in detail in our manuscript. Unlike flu databases with full length sequences, for example, partial VP1 sequences dominates repositories such as Genebank and as such our efforts to determine temporal events and genetic shifts were not successful (line 318 to 336 for detail discussion). We believe this highlight a need for full genome sequences of enteroviruses going forward as much information is lost by partial gene sequencing.**

Reviewer's Responses to Questions

Methods

-Are the objectives of the study clearly articulated with a clear testable hypothesis stated?</br> -Is the study design appropriate to address the stated objectives?</br> -Is the population clearly described and appropriate for the hypothesis being tested?</br> -Is the sample size sufficient to ensure adequate power to address the hypothesis being tested?</br> -Were correct statistical analysis used to support conclusions?</br> -Are there concerns about ethical or regulatory requirements being met?</br></br>

Reviewer #1: This is a retrospective studies for the surveilance study of HFMD in Singapore.

The enrolled patient was appropriate for this objective.

**We would like to thank the reviewer for saying “The enrolled patient was appropriate for this objective”.**

Reviewer #2: Since authors have just describe the genotype of Enterovirus for HFMD. The term of "cohort" in Methods is not right. Actually, authors have not described the design following the guideline of cohort study. The design of the present study is open to question. We could take it as a surveillance for HFMD epidemic in Singapore. Considered that, it is better to rewrite the Methods. I am confused the description of the present study. More work is need to make it clear.

**We would like to thank the reviewer for the point raised.** **We studied possible factors such as age, gender, race and prior HFMD history to determine whether they have potential to be a risk factor for symptomatic and asymptomatic HFMD in Singapore at the time period of 2013-2018. As such, we retrospectively identified those factors and “prospectively” compared them between healthy, symptomatic and asymptomatic cohorts as potential risk factor for HFMD which in our humble opinion could be seen as a retrospective cohort study. However, we accept the reviewer point that in a way this study could also be identified as a surveillance study for HFMD as molecular epidemiology aspect of endemic HFMD in Singapore were delved into as well. As such we have made changes to the title as follow: “An Epidemiological Surveillance of Hand Foot and Mouth Disease in Paediatric Patients and in Community: A Singapore Retrospective Cohort Study, 2013-2018”**

Reviewer #3: (No Response)

Results

-Does the analysis presented match the analysis plan?</br> -Are the results clearly and completely presented?</br> -Are the figures (Tables, Images) of sufficient quality for clarity?</br></br>

Reviewer #1: They genotyped 101 HFMD infected samples and demonstrated that CV-A6 is the major etiological virus for the outbreaks during 2013 to 2018 in Singapore.

The short sequence of VP1 was used to compare the local CV-A6 samples in Singapore against those globally, however, the dominant genetic lineage of CV-A6 viruses found in Singapore coincide with viruses that were found globally. The conclusion is not clear.

All of the figures are complex and not easy to understand

**We would like to thank the reviewer for the points raised. HFMD is endemic and the incidence rate is around 6%–14% at age 1 in Singapore which is considered relatively high in the region [2]. We found 3 lineages of CV-A6 circulating in Singapore (Figure 3A) and the major lineage consist of CV-A6 strains, which are found to be circulating globally and also closely related to strains from Asia such as Thailand, Malaysia and Japan (Figure 3B).**

Reviewer #2: 1.Only over 100 samples collected from 2013 to 2018, why did authors mentioned "recent outbreaks" in Abstract?

**There were several hundreds of HFMD hospitalized cases in Singapore typically in a given year. However, recruitment of patient for consent so samples could be used in medical research was proved to be extremely challenging throughout the study. We postulate that it could be due to several reasons including the age of patient, guardians not willing to disclose personal data outside of the hospital and guardian(s) refusing to participate due to the lethargic nature of the disease inflicted on patient.**

2.The characteristics of patients and health controls should be shown in one table. Are there any change of them from 2013 to 2018?

**We would like to thank the reviewer for the points raised. The data are collected from two different locations and as such segregated into the two tables; Table 1 is from KK Women Hospital and Table 2 consist of data collected routinely from childcare centers.**

3.Figure 5 is questionable. Why are the data from 2012 included in the figure? What is the meaning of Figure 5B? Is it the data from 2010 to 2018? Are there any time trend for it? Figure 5A and 5C could be shown in a table.

**We thank the reviewer for the comment. The typo has been corrected from 2012 to 2013. Figure 5B depicts the recalled frequency of individual clinical features from asymptomatic HFMD patients during their previous HFMD episodes.**

Reviewer #3: In page 8 line 155, understandably a full recovery means no symptoms and fever present. However, does the partial recovery status mean that some of the symptoms such as mouth ulcers or rashes are still present or just a slight fever?

**This question has been addressed above.**

Page 22. Please clarify the grading for characteristics in Table 2.

**This question has been addressed above.**

Page 27 Figure 3. The text in this figure is difficult to distinguish, even in the PDF file. Is there a clearer figure to replace this one?

**This question has been addressed above.**

--------------------

<b>Conclusions</b></br></br>

-Are the conclusions supported by the data presented?</br> -Are the limitations of analysis clearly described?</br> -Do the authors discuss how these data can be helpful to advance our understanding of the topic under study?</br> -Is public health relevance addressed?</br></br>

Reviewer #1: The conclusion is clear and well addressed.

The authors should try to determine the temporal event of CV-A6 genetic shifts in the past while comparing the local sequence with global strains, however, as most of the CV-A6 sequences in GeneBank were only partial VP1 sequences leading to a highly diffuse temporal signal.

The author raise the silent Enterovirus infections in this study, indicating that spread of subclinical HFMD is happened.

**We would like to thank the reviewer for saying “The conclusion is clear and well addressed.”**

Reviewer #2: The conclusion seems to be supported by the data presented. But we need more details from the study.

**We would like to thank the reviewer for saying “The conclusion seems to be supported by the data presented.”**

Reviewer #3: (No Response)

--------------------

<b>Editorial and Data Presentation Modifications?</b></br><br/> Use this section for editorial suggestions as well as relatively minor modifications of existing data that would enhance clarity. If the only modifications needed are minor and/or editorial, you may wish to recommend “Minor Revision” or “Accept”.

Reviewer #1: For Abstract: “We studied symptomatic paediatric HFMD cases from 2013 to 2018 in Singapore. Surveillance for subclinical enterovirus infections was also performed in childcares at the same time period.” At least, please describe how to detect the different types of Enteroviruses and sequence and Phylogenetic analysis.

**We would like to thank the reviewer for the input. Due to the strict word limit we were not able to describe the detection method and analytical methods in detail in the abstract. However, such methodologies and reasonings were detailed in Method section, “Detection of Enteroviruses” from line 126-147.**

Reviewer #2: (No Response)

Reviewer #3: Page 9 line 188. A typo was written as ‘throat-swan’.

**This question has been addressed above.**

Page 10 line 194. The abbreviation for CV-A16 was used inconsistently and was written as CA16.

**This question has been addressed above.**

Page 11 Line 225. Reference style inconsistency.

**This question has been addressed above.**

--------------------

<b>Summary and General Comments</b></br></br> Use this section to provide overall comments, discuss strengths/weaknesses of the study, novelty, significance, general execution and scholarship. You may also include additional comments for the author, including concerns about dual publication, research ethics, or publication ethics. If requesting major revision, please articulate the new experiments that are needed.

Reviewer #1: In this study, Min et al., reported that CV-A6 is the major etiological 38 agents for the recent HFMD outbreaks during 2013 to 2018 after genotyping 101 symptomatic HFMD samples in Singapore. They also detected sub-clinical enterovirus infections in childcare centres and revealed that 17.1% (n=245) tested positive for enterovirus in saliva, without HFMD indicative symptoms at the same period. This is an interesting and important information for the Enteroviruses surveillance in Singapore. Also, the results indicate the subclinical cases in Singapore and HFMD outbreaks often occurs sporadically and frequently in the childcare and other children’s learning institutions.

There is only minor revision should be addressed:

Keywords: “Singapore” should not be as a keyword

**We thank the reviewer for the kind comments. Keyword Singapore has been removed.**

As for Figure 1A, I notice that there is CA-6 (36%, brown color) shown in the positive region and addition CA-6 (8% in blue color) shown in the overlap region, can the authors explain this result?

**We thank the reviewer for clarification. The 36% is detected with partial VP1 sequencing and 8% is confirmed with next generation sequencing. The information is written at the bottom of the respective arc.**

Line 307-308: “VP1 sequencing was favoured for genotyping of enteroviruses since the development for partial VP1 sequencing primers and as such 333bp sequences dominated the GenBank”, please revised the sentence as the following:

Due to the development of some VP1 sequencing primers and the 333bp sequence dominating GenBank, VP1 sequencing is favored in enterovirus genotyping..

**We thank the reviewer for clarification. Changes has been made to the sentence.**

Reviewer #2: Since authors want to describe the change by years, more work is need to illustrate that. I do not think the present study has been well reported. The desing and data presented are also questionable. More work are need to make the outline clear.

**We thank the reviewer for the comment. We have attempted to detect genetic shifts and temporal changes across the years in this study but the attempt was not successful due to inherent nature of VP1 sequences deposited in the gene bank along with highly uneven and inadequate sampling of CV-A6 viruses worldwide resulting in poor molecular clock signal (Figure 4). We have discussed this in the manuscript from line 318 to 336.**

Reviewer #3: The authors of this manuscript studied HFMD and subclinical cases from the years 2013 to 2018 in Singapore. Their findings showed that majority of the samples collected were cased by coxsackievirus A6, while a smaller percentage being caused by EV-A71, CV-A16, CV-A2, and CV-A10. They also compared the positive testing rate between samples collected via throat swab and from saliva. The findings indicated that samples from throat swabs had a higher positive rate in virus detection compared to saliva samples. The authors also reported that 17.1% tested positive for enterovirus in saliva among healthy children in daycare centers.

**We would like to thank the reviewer for the comprehensive summary.**

--------------------

PLOS authors have the option to publish the peer review history of their article (<a href="https://journals.plos.org/plosntds/s/editorial-and-peer-review-process#loc-peer-review-history" target="_blank">what does this mean?</a>). If published, this will include your full peer review and any attached files.<br><br>

If you choose “no”, your identity will remain anonymous but your review may still be made public.<br><br>

<b>Do you want your identity to be public for this peer review?</b> For information about this choice, including consent withdrawal, please see our <a href="https://www.plos.org/privacy-policy" target="_blank">Privacy Policy</a>.

Reviewer #1: No

Reviewer #2: Yes: Sheng Wei

Reviewer #3: Yes: Jen-Ren Wang

**Refrences**

1.	Cardosa MJ, Perera D, Brown BA, Cheon D, Chan HM, Chan KP, et al. Molecular Epidemiology of Human Enterovirus 71 Strains and Recent Outbreaks in the Asia-Pacific Region: Comparative Analysis of the VP1 and VP4 Genes. Emerg Infect Dis. 2003;9(4):461-8. doi: 10.3201/eid0904.020395.

2.	Koh WM, Badaruddin H, La H, Chen MI-C, Cook AR. Severity and burden of hand, foot and mouth disease in Asia: a modelling study. BMJ Global Health. 2018;3(1). doi: 10.1136/bmjgh-2017-000442.
